# Analgesic Strategies in Adult Intensive Care Units: A Systematic Review of Opioid and Non-opioid Approaches

**DOI:** 10.7759/cureus.101790

**Published:** 2026-01-18

**Authors:** Majed M Madkhali, Hussain M Al Qibti, Mohammed A Al-Arim, Muaid A Alfaifi, Maram M Fageehi, Ohoud M Masmali, Fawziah M Jali, Abdulelah Y Beati, Leena M Almobty, Moath A Alqanbar, Shaima M Alabdullah, Khaled A Hakami

**Affiliations:** 1 Pediatric Anesthesiology, Prince Mohammed bin Nasser Hospital, Jazan, SAU; 2 Internal Medicine, King Fahad Central Hospital, Jazan, SAU; 3 General Practice, College of Medicine, Jazan University, Jazan, SAU; 4 General Practice, College of Medicine, King Khalid University, Abha, SAU; 5 General Practice, College of Medicine, Alahssa University, Alahssa, SAU; 6 General Practice, College of Medicine, King Faisal University, Hofuf, SAU; 7 General Practice, King Fahad Central Hospital, Jazan, SAU

**Keywords:** analgesia-first sedation, critical care, dexmedetomidine, intensive care unit, light sedation, multimodal analgesia, non-opioid analgesics, opioid-sparing strategies, protocolized sedation, remimazolam

## Abstract

Effective analgesia and sedation are critical components of care in adult intensive care units, with strategies ranging from opioid and non-opioid regimens to multimodal and protocol-guided approaches. This systematic review was conducted in accordance with Preferred Reporting Items for Systematic Reviews and Meta-Analyses (PRISMA) guidelines. Evidence was synthesized from randomized controlled trials and observational studies across PubMed, Cochrane Library, Scopus, and Web of Science through October 2025, evaluating sedative and analgesic interventions - including opioid, non-opioid, alpha-2 agonist, and protocolized approaches - in adult intensive care unit (ICU) populations. The methodological quality of the chosen studies was assessed using the Modified Downs and Black checklist.

A total of 11 eligible studies demonstrated that dexmedetomidine provided comparable or superior sedation quality relative to benzodiazepines and propofol, reduced delirium incidence, and shortened time to extubation; remimazolam yielded equivalent sedation to propofol with fewer hypotensive events; and protocolized or nurse-driven sedation strategies improved ventilator-free days and reduced ICU length of stay compared with daily interruption alone. Multimodal and non-opioid adjuvant therapies reduced opioid requirements and nausea while maintaining hemodynamic stability, and across studies, light, cooperative sedation paired with validated pain and sedation assessment tools was associated with better recovery profiles and fewer cognitive complications.

Overall, the evidence supports adopting multimodal, analgesia-first, and non-gamma-aminobutyric-acid (non-GABA) sedation approaches in critical care, with dexmedetomidine and remimazolam offering superior safety and delirium prevention compared with benzodiazepines.

## Introduction and background

Effective analgesia and sedation are central to modern intensive care, shaping physiological stability, patient comfort, and clinical outcomes. Pain, agitation, and delirium frequently coexist in the intensive care unit (ICU), contributing to prolonged mechanical ventilation and increased morbidity [1,2]. Despite the availability of structured guidelines, substantial variation in practice persists, reflecting ongoing uncertainty about the optimal use of different analgesic and sedative agents [1].

Benzodiazepines and opioids have long been the foundation of ICU sedation and analgesia, yet prolonged exposure to these agents is associated with delirium, respiratory depression, and delayed recovery [3-10]. These concerns have driven a shift toward minimizing deep sedation and promoting early awakening, leading to increasing interest in newer sedatives and multimodal approaches that aim to provide cooperative sedation with fewer neurocognitive and hemodynamic complications [3-7,9].

Recent clinical trials have evaluated alternative agents and protocolized strategies designed to individualize analgesia and sedation. Agents such as dexmedetomidine, propofol, and remimazolam have been studied for their potential to reduce delirium and facilitate earlier extubation [3-9]. At the same time, nurse-driven algorithms, daily sedation interruption, and multimodal analgesic regimens incorporating non-opioid adjuvants have been adopted to reduce oversedation and limit opioid exposure [2].

However, evidence across studies remains heterogeneous, with inconsistent findings regarding effects on delirium, mechanical ventilation duration, ICU length of stay, and other key outcomes [1,3-10]. Differences in study design, patient characteristics, sedation targets, and outcome definitions further challenge interpretation and implementation in clinical practice [9]. Important gaps also remain in understanding how these pharmacologic strategies interact with the ICU environment, illness severity, and concurrent therapies [11].

Given these uncertainties, a comprehensive synthesis of current evidence is needed to clarify the relative effectiveness and safety of opioid and non-opioid analgesic and sedative strategies in critically ill adults. This systematic review evaluates their impact on pain control, delirium, sedation quality, duration of mechanical ventilation, recovery outcomes, and adverse events, to inform evidence-based clinical practice and identify priorities for future research [1-11].

## Review

Methods

Literature Search Strategy

This systematic review followed the Preferred Reporting Items for Systematic Reviews and Meta-Analyses (PRISMA) guidelines [12]. A comprehensive search of PubMed, the Cochrane Library, Web of Science, and Scopus was conducted to identify studies evaluating analgesic and sedative strategies in adult ICU patients (Appendix). Searches included all available literature up to October 2025 and focused on outcomes such as pain control, delirium, sedation depth, duration of mechanical ventilation, recovery, and mortality.

The search strategy incorporated both Medical Subject Headings (MeSH) and free-text terms related to analgesia, sedation, critical care, adult populations, and common clinical outcomes. Equivalent search structures were adapted for each database. Searches were limited to English-language human studies and restricted to original research, including randomized controlled trials (RCTs), controlled clinical trials, and observational studies.

Eligibility Criteria

Study eligibility was defined using the Population-Exposure-Comparator-Outcome (PECO) framework [13]. Eligible studies included adults admitted to an ICU for medical or surgical conditions requiring analgesia, sedation, or mechanical ventilation. Exposures of interest included opioid-based or non-opioid regimens such as dexmedetomidine, propofol, benzodiazepines, haloperidol, remimazolam, and multimodal strategies. Studies were required to include a comparator group and report at least one clinically relevant outcome, including pain ratings, delirium incidence or duration, sedation adequacy, ventilator-free days, length of stay or mortality.

Eligible designs included RCTs, controlled clinical trials, and prospective observational studies. Studies were excluded if they involved pediatric populations; focused solely on perioperative or procedural sedation; lacked relevant clinical outcomes; or were review articles, case reports, conference abstracts, editorials, or animal studies. Only full-text publications in English were included.

Study Selection

All identified records were imported into EndNote (Clarivate, Philadelphia, PA) for organization and duplicate removal. Study selection occurred in two stages. First, two reviewers independently screened titles and abstracts based on the inclusion and exclusion criteria. Second, full-text articles of potentially eligible studies were reviewed in detail. Disagreements were resolved through discussion or consultation with a third reviewer.

Data Extraction and Quality Appraisal

Two reviewers independently extracted data using a standardized form. Extracted variables included study characteristics, patient demographics, type and dose of interventions, comparator regimens, and reported clinical outcomes such as sedation adequacy, delirium, mechanical ventilation duration, pain control, and recovery indicators. Differences in extraction were resolved by consensus, with third-reviewer arbitration when needed.

Methodological quality and risk of bias were assessed using the Downs and Black Checklist, which evaluates reporting quality, external validity, internal validity, and study power [14]. Studies were rated as excellent, good, fair, or poor based on their total score. Most included studies demonstrated high methodological quality, supporting reliable interpretation and synthesis of findings.

Results

Study Selection

**Figure 1:**
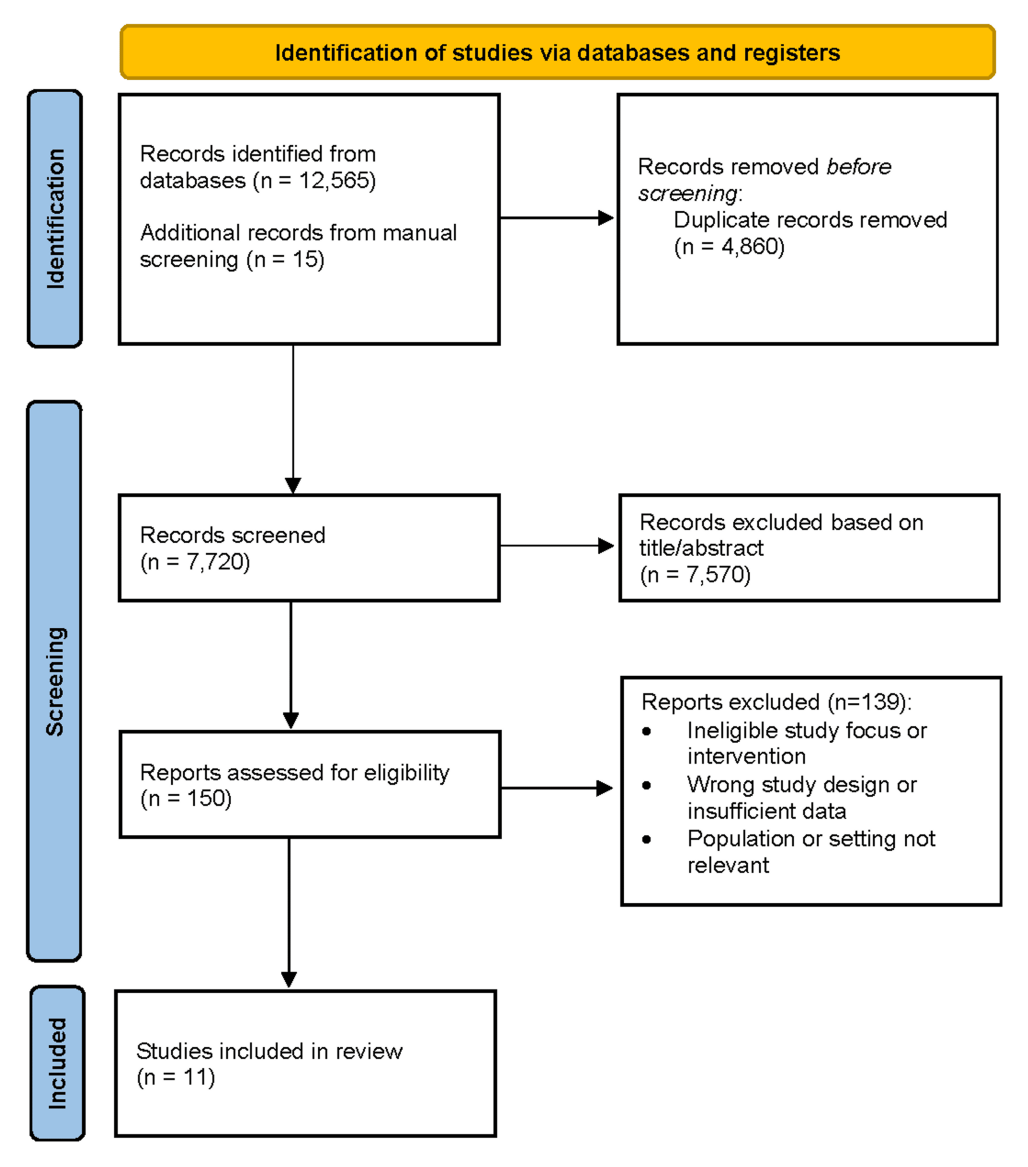
PRISMA flow diagram of the study selection process The flow diagram summarizes the selection of studies for inclusion in the systematic review following PRISMA recommendations [12]. It details the number of records identified, screened, and assessed for eligibility, as well as the studies included in the final analysis, with exclusions at each stage and corresponding reasons documented.

Database searches identified 12,580 records, with an additional 15 records found through manual screening. After removal of duplicates, 7,720 unique studies were assessed by title and abstract, and 7,570 were excluded as irrelevant. Full texts of 150 articles were evaluated, resulting in 11 studies [1-11] that met all inclusion criteria. These studies demonstrated acceptable methodological quality and were included in the qualitative synthesis. The selection process is summarized in the PRISMA flow diagram (Figure 1).

Characteristics of Included Studies

The 11 included studies consisted of nine RCTs, one prospective single-center trial, and one multicenter observational study conducted across multiple continents. Sample sizes ranged from 71 to 700 patients. Most studies enrolled adults requiring mechanical ventilation or postoperative monitoring in mixed medical-surgical ICUs, while others focused on subgroups such as sepsis [5], agitated delirium [4,7], or organ dysfunction [6]. Methodological rigor was generally high, with defined inclusion criteria, standardized sedation targets, and validated measures for sedation, delirium, and recovery (Table 1).

**Table 1:** Summary of characteristics of the included studies Summary of RCTs and observational studies evaluating sedation strategies in critically ill adult patients in ICUs. The table includes study author, country/setting, design, sample size, population characteristics, interventions and comparators, outcomes measured, key findings/results, and safety/adverse events. Interventions include sedatives such as dexmedetomidine, propofol, remimazolam, midazolam, lorazepam, haloperidol, and protocols such as DIS and nurse-driven SAs. Outcomes include sedation effectiveness measured by RASS, delirium incidence assessed by CAM-ICU or ICDSC, VFDs, DCFDs, ICU/LOS, MV duration, organ function (SOFA score), pain (NRS), and patient safety measures. Adverse events reported include bradycardia, hypotension, tachycardia, accidental device removal, and other sedation-related complications. Abbreviations: ICU = intensive care unit; APACHE: Acute Physiology and Chronic Health Evaluation; MV = mechanical ventilation; NMBD = neuromuscular blocking drug; DIS = daily interruption of sedation; AE = adverse event; RCT = randomized controlled trial; TBI = traumatic brain injury; NM = neuromuscular; LOS = length of stay; ICDSC = Intensive Care Delirium Screening Checklist; VAS = visual analogue score; DEX = dexmedetomidine; CAM-ICU = Confusion Assessment Method for ICU; NRS = Numeric Rating Scale; NS = non-significant; RASS = Richmond Agitation-Sedation Scale; MAAS = Motor Activity Assessment Scale; VFDs = ventilator-free days; SOFA = Sequential Organ Failure Assessment; CRP = C-reative protein; DCFDs = delirium- and coma-free days; QoL = quality of life; BP = blood pressure; DIS = daily interruption of sedation; SA = sedation algorithm

Author	Country / Setting	Study Design	Sample Size	Population Characteristics	Intervention (Type, Dose, Duration)	Comparator	Outcomes Measured	Key Findings / Results	Safety / Adverse Events
Burry et al. [1]	Canada / 51 ICUs	Prospective, multicenter observational study	712 mechanically ventilated adults (3,620 patient-days)	Critically ill adults on MV ≥24 h; median age 61; APACHE II ≈19.6; mixed medical-surgical	Observational data collection; recorded opioid, sedative, NMBD, antipsychotic use; sedation/pain/delirium assessment tools, protocols, DISs	None	Frequency/type/route of analgesics, sedatives, antipsychotics, NMBDs; secondary: assessment tool and protocol use, predictors	Opioid use 84.8% (fentanyl 54.3%, morphine 35%), sedatives 62.2% (midazolam most), antipsychotics 9.6%, NMBDs 18.3%, DIS 42%; predictors identified for drug/protocol use	Accidental device removal 4.6%; 75% during DIS; no major sedation-related AEs
Mehta et al. [2]	Canada & USA / 16 tertiary ICUs	Multicenter RCT (SLEAP)	423 analyzed (214 DIS + protocolized sedation, 209 protocolized sedation)	Mechanically ventilated adults expected ≥48 h; excluded cardiac arrest, TBI, NM blockade, palliative intent	Protocolized sedation + DIS: opioid/benzodiazepine titration to light sedation, daily stops	Protocolized sedation only	Time to successful extubation, ICU/hospital LOS, sedative/opioid doses, delirium (ICDSC ≥4), unintentional device removal, mortality, nurse workload	Median time to extubation 7 days both; ICU/hospital LOS similar; delirium ~53%; sedative/opioid use higher with DIS; nurse workload higher (VAS 4.22 vs 3.80, p=0.001)	Unintentional device removal similar (4.7% vs 5.8%); no increase in serious AEs; more agitation and sedative requirement with DIS
Su et al. [3]	China / Peking University First & Third Hospitals	Randomized, double-blind, placebo-controlled clinical trial	700 (350 DEX, 350 placebo)	Adults ≥65 yrs ICU post non-cardiac surgery; excluded neuro disorders, severe cardiac dysfunction, dialysis, coma	DEX 0.1 µg/kg/h IV continuous (~14–16 h); no loading dose	Placebo (normal saline)	Delirium incidence (CAM-ICU, days 1–7), time to extubation, ICU/hospital LOS, complications, 30-day mortality, pain (NRS), sleep, hemodynamics	Delirium 9% vs 23% (OR 0.35, p<0.0001); time to extubation 4.6 vs 6.9 h; ICU LOS 20.9 vs 21.5 h; non-delirium complications 15% vs 21%; improved pain and sleep (all p<0.001)	Bradycardia 17% vs 13% NS; hypotension 33% vs 26% NS; tachycardia lower (7% vs 14%, p=0.002); hypertension and hypoxemia lower; no sedation-related serious events
Riker et al. [4]	Multinational / 68 ICUs (USA, Australia, Argentina, Brazil, New Zealand)	Prospective, double-blind RCT (SEDCOM)	366 analyzed (244 DEX, 122 midazolam)	Adult medical/surgical ICU patients expected MV >24 h; mean age 62; APACHE II ≈19; 75% sepsis; excluded trauma, pregnancy, severe hepatic/cardiac disease	DEX 0.2–1.4 μg/kg/h IV up to 30 days; titrated to RASS −2 to +1; optional loading 1 μg/kg; fentanyl PRN	Midazolam 0.02–0.1 mg/kg/h IV; titrated to the same sedation target; fentanyl PRN	% time within RASS target, delirium prevalence/duration, MV duration, ICU LOS, nurse interaction, 30-day mortality	Sedation similar (77.3% vs 75.1%); delirium lower with DEX (54% vs 76.6%); time to extubation 3.7 vs 5.6 days; delirium-free days higher; communication improved	Bradycardia 42% vs 19% (5% required intervention); tachycardia less frequent; hypertension needing treatment less; infections lower; no rebound HTN or severe withdrawal
Jakob et al. [5]	Europe / 44 ICUs (MIDEX), 31 ICUs (PRODEX)	Two multicenter, double-blind, phase 3 RCTs	MIDEX: 500 (249 Dex, 251 Midazolam); PRODEX: 498 (251 Dex, 247 Propofol)	Mechanically ventilated adults needing light–moderate sedation >24 h; excluded severe neuro disorders, bradycardia <50 bpm, high vasopressor need	DEX 0.2–1.4 µg/kg/h IV continuous; daily sedation stops, spontaneous breathing trials	MIDEX: Midazolam 0.03–0.2 mg/kg/h IV; PRODEX: Propofol 0.3–4 mg/kg/h IV	% time within target RASS, duration of mechanical ventilation, ICU/hospital LOS, pain communication, arousability (VAS), delirium (CAM-ICU), 45-day mortality	Sedation efficacy: Dex non-inferior; ventilation shorter vs midazolam (123 vs 164 h, p=0.03); extubation shorter vs both; improved patient communication; lower neurocognitive events vs propofol	Bradycardia higher with DEX (14% vs 5% midazolam, 3.7% vs 0.8% propofol); hypotension was higher vs. midazolam (20.6% vs 11.6%), similar vs. propofol; no increase in serious AEs overall
Reade et al. [6]	Australia and New Zealand / 15 ICUs	Double-blind, multicenter RCT (DahLIA)	71 analyzed (39 DEX, 32 placebo)	Mechanically ventilated adults with agitated delirium (CAM-ICU +, MAAS ≥5), ready for extubation but restrained; excluded dementia, TBI, α₂-agonist therapy	DEX 0.5 μg/kg/h IV, titrated 0–1.5 μg/kg/h to RASS −2 to +1; max 7 days; optional bolus	Placebo (normal saline); standard sedation/antipsychotics per clinician	Ventilator-free hours within 7 days, time to extubation, delirium resolution, ICU/hospital LOS, antipsychotic/sedative use, mortality	Ventilator-free hours 144.8 vs 127.5 h (Δ=17 h, p=0.01); time to extubation 21.9 vs 44.3 h; delirium resolution 23.3 vs 40 h; antipsychotic use less; opioid/propofol use reduced	Bradycardia 5% vs 0%; mild hypotension; rare agitation-related AEs; no increase in reintubation/self-extubation
Kawazoe et al. [7]	Japan / 8 ICUs	Open-label, multicenter RCT (DESIRE Trial)	201 (100 DEX, 101 Control)	Adult ICU patients with sepsis requiring MV ≥24 h; mean age 69 ± 14 yrs; 63% male; median APACHE II 23; 71% shock on admission	DEX 0.2–1.4 µg/kg/h IV; target RASS 0 (day) to −2 (night); additional agents as needed	No DEX; sedation with midazolam, propofol, fentanyl per protocol	28-day mortality, ventilator-free days (VFDs), SOFA, ICU LOS, delirium/coma-free days, sedation quality, CRP, procalcitonin, renal outcomes	28-day mortality 22.8% vs 30.8% (HR 0.69, p=0.20); VFDs 20 vs 18; sedation improved with DEX (17–58% vs 20–39%, p=0.01); subgroup APACHE II ≥23: lower mortality (HR 0.39, p=0.03)	Bradycardia 7% vs 2% (NS); mild hypotension; ACS 1% both; no serious sedation-related events
Pandharipande et al. [8]	USA / Vanderbilt and Washington Hospital Center	Double-blind RCT (MENDS trial)	103 analyzed (52 DEX, 51 lorazepam)	Adult medical/surgical ICU patients needing MV >24 h; median age 60; APACHE II 29; excluded severe neuro, alcohol withdrawal, hepatic failure	DEX 0.15–1.5 µg/kg/h IV up to 120 h; no loading dose	Lorazepam 1–10 mg/h IV	DCFDs, % time at target RASS, ventilator-free days, ICU/hospital LOS, 28-day and 12-month mortality, neurocognitive outcomes, costs	DCFDs 7 vs 3 (p=0.01); coma prevalence 63% vs 92%; target sedation 80% vs 67%; 28-day mortality 17% vs 27%	Bradycardia 17% vs 4% (one treated); hypotension similar; self-extubation 8% vs 4%; no hemodynamic compromise or severe toxicity
He et al. [9]	China / Surgical ICU, Third Affiliated Hospital of Sun Yat-Sen University	Prospective single-center RCT	120 (60 per group)	Adult ICU patients (18–80 yrs) with hepatic or renal dysfunction requiring fiberoptic bronchoscopy	Remimazolam besylate (initial 5 mg IV; 2.5 mg q15min × ≤5 if needed)	Propofol (1.5 mg/kg IV load; 0.5 mg/kg q15min × ≤5 if needed)	Sedation success, time to target sedation (RASS −3 to 0), recovery time, hemodynamic events, liver/renal function	Both groups achieved target sedation in ~5 min; remimazolam required fewer rescue doses (3.3% vs 16.7%, p=0.03); cumulative sedative dose was lower (5 mg vs 100 mg, p<0.001); recovery time was similar	Hypotension 3.3% vs 20% (p=0.01); bradycardia, tachycardia, hypoxemia not different; no severe adverse events
Smit et al. [10]	Netherlands / 8 mixed medical–surgical ICUs	Multicenter, double-blind, placebo-controlled RCT	132 (65 haloperidol, 67 placebo)	Adult ICU patients (mean 64 ± 15 yrs, 68% male) with delirium (CAM-ICU + or ICDSC ≥4); excluded neurological disease, dementia, psychosis	Haloperidol IV 2.5 mg q8h (1 mg if ≥80 yrs), titrated up to 5 mg q8h; duration up to 14 days	Placebo (IV saline)	Delirium- and coma-free days (DCFDs), secondary: sedative/antipsychotic use, agitation, self-extubation, mobility, sleep, 28-day mortality, post hoc: falls, hallucinations, memory, QoL	No difference in DCFDs (9 vs 9, aRR 0.98, p=0.87); haloperidol had less benzodiazepine use (57% vs 73%, aOR 0.41, p=0.03); trends toward fewer agitation events and falls (9% vs 27%, aOR 0.32, p=0.03)	Adverse events rare; QTc prolongation 5% vs 9%, arrhythmia 6% vs 2%, NS; slight BP drop after first dose (−12 mmHg, p=0.001); no extrapyramidal differences
de Wit et al. [11]	USA / Virginia Commonwealth University Medical Center, Medical ICU	Prospective RCT	74 (36 DIS, 38 SA)	Adult mechanically ventilated ICU patients; excluded severe neurocognitive dysfunction, tracheostomy, NM blockade	DIS until awake/agitated; resumed at half prior dose	SA: nurse-driven sedation algorithm (RASS −2 to −3)	Total duration of mechanical ventilation, 28-day ventilator-free days, ICU/hospital LOS, SOFA, sedation depth, agitation, sedative/analgesic use, mortality	SA reduced mechanical ventilation (3.9 vs 6.7 days, p=0.0003), ICU LOS (8 vs 15 days, p<0.0001), hospital LOS (12 vs 23 days, p=0.01); more ventilator-free days; DIS group had more agitation	Early hypertension, tachycardia, patient–ventilator asynchrony during DIS; more withdrawals due to agitation; no major drug-related AEs

Interventions encompassed pharmacologic regimens and protocolized sedation strategies. Dexmedetomidine was evaluated in six major trials [3,4,6-8], and remimazolam in one study [9], typically compared with benzodiazepines or propofol under light-to-moderate sedation targets assessed by the Richmond Agitation-Sedation Scale (RASS). One study assessed haloperidol, an antipsychotic medication [10], and two examined protocol-based sedation: a nurse-implemented algorithm versus daily interruption [11] and protocolized sedation with or without daily interruption [2]. An observational study described real-world sedative, opioid, and antipsychotic use across Canadian ICUs [1].

Outcome measures included sedation quality, delirium incidence or duration, ventilator-free days, ICU or hospital length of stay, and adverse events [2,5]. Dexmedetomidine and remimazolam generally produced effective light sedation with favorable hemodynamic and cognitive profiles compared with γ-aminobutyric acid (GABA)-ergic sedatives [7,9]. Antipsychotics modestly reduced agitation without shortening delirium duration, while algorithm-based sedation shortened ventilation and ICU stay compared with daily interruption. Reported adverse events were mostly mild and related to drug dosing [2-8]. Overall, the included studies reflect a shift toward individualized, non-opioid, and protocol-guided sedation strategies aimed at reducing delirium and accelerating recovery [2-8].

Quality Assessment

Study quality assessed using the Modified Downs and Black Checklist ranged from 24 to 28, indicating good to excellent methodological rigor. Eight studies achieved an excellent rating, and three were rated good. Reporting quality was consistently high, with clear intervention descriptions and outcome definitions. External validity was strongest in large multicenter trials enrolling diverse ICU populations. Internal validity was robust in double-blind RCTs such as those by Su et al. [3], Jakob et al. [5], and Pandharipande et al. [8], whereas open-label designs [1,6] had moderate confounding risk. All studies demonstrated adequate statistical power with appropriate sample size calculations and prespecified endpoints, supporting confidence in the reliability of findings (Table 2).

**Table 2:** Summary of the quality appraisal of the included studies Quality assessment of included RCTs and observational studies using the Downs and Black checklist [14]. Reporting scores (0–10) reflect completeness and transparency of study reporting; External Validity (0–3) assesses generalizability to broader populations; Internal Validity – Bias (0–7) evaluates potential for systematic errors affecting study results; Internal Validity – Confounding (0–6) reflects adequacy of controlling for confounders; Power (0–2) indicates whether sample size and statistical power were sufficient; Total (0–28) represents the cumulative quality score. Quality Level categorizes studies as Excellent, Good–Excellent, or Good based on the total score. Notes/Comments summarize methodological strengths, limitations, and context of the findings. Abbreviations: RCT = randomized controlled trial; ICU = intensive care unit; CAM-ICU = confusion assessment method for the ICU; EuRIDICE = European randomized ICU delirium clinical evaluation; SAS = sedation–agitation scale; RASS = Richmond Agitation–Sedation Scale; DIS = daily interruption of sedation; NMBD = neuromuscular blocking drug; VAS = visual analog scale; SLEAP = sedation practice in intensive care evaluation; MENDS = maximizing efficacy of targeted sedation; DahLIA = Dexmedetomidine for agitated delirium in the ICU; and SEDCOM = safety and efficacy of dexmedetomidine compared with midazolam.

Author	Reporting (0–10)	External Validity (0–3)	Internal Validity – Bias (0–7)	Internal Validity – Confounding (0–6)	Power (0–2)	Total (0–28)	Quality Level	Notes / Comments
Burry et al. [1]	9	3	6	4	2	24 / 28	Good	Prospective observational multicenter Canadian ICU study. Strong data quality and generalizability; limited by observational design and potential confounding.
Mehta et al. [2]	10	3	7	6	2	28 / 28	Excellent	SLEAP multicenter RCT of DIS + protocolized sedation. Strong internal validity, validated tools, and high statistical power.
Su et al. [3]	10	3	7	6	2	28 / 28	Excellent	Large multicenter double-blind RCT; clear randomization, power analysis, and outcome definition. Reduced delirium observed with dexmedetomidine.
Riker et al. [4]	10	3	7	6	2	28 / 28	Excellent	SEDCOM multicenter double-blind RCT (dexmedetomidine vs midazolam). Perfect reporting; large sample; validated outcomes; minimal bias.
Jakob et al. [5]	10	3	7	6	2	28 / 28	Excellent	MIDEX/PRODEX phase-3 RCTs of dexmedetomidine vs midazolam/propofol. Methodologically outstanding; high power and strong internal validity.
Reade et al. [6]	10	3	7	5	2	27 / 28	Excellent	DahLIA double-blind RCT adding dexmedetomidine to standard care for agitated delirium. High rigor; early stop slightly reduced power.
Kawazoe et al. [7]	10	3	6	5	2	26 / 28	Good–Excellent	DESIRE open-label multicenter RCT in septic ICU patients. Robust design and outcomes but lack of blinding slightly reduces bias control.
Pandharipande et al. [8]	10	3	7	6	2	28 / 28	Excellent	MENDS double-blind RCT (dexmedetomidine vs lorazepam). Excellent design, blinding, and validated delirium metrics (CAM-ICU).
He et al. [9]	10	3	7	5	2	27 / 28	Excellent	High-quality RCT of remimazolam vs propofol for ICU bronchoscopy. Excellent reporting and design; minor limitation: no adjusted multivariate analysis.
Smit et al. [10]	10	3	7	6	2	28 / 28	Excellent	EuRIDICE RCT of haloperidol for ICU delirium. Double-blind, multicenter, rigorous protocol. Early termination slightly reduced power, but internal validity remained strong.
de Wit et al. [11]	9	2	6	5	2	24 / 28	Good	RCT comparing DIS vs nurse-driven algorithm. Well-reported but single-center, small sample, moderate generalizability.

Results on Pain and Sedation Quality

Both opioid and non-opioid regimens achieved effective sedation, but notable differences emerged in interaction quality and depth control. Dexmedetomidine consistently produced comparable or superior sedation to midazolam and propofol, with better communication and fewer episodes of coma or oversedation in SEDCOM and MIDEX/PRODEX [4,5], and more coma-free days than lorazepam in Pandharipande et al. [8]. Remimazolam provided equivalent sedation to propofol with faster onset, lower cumulative dose, and fewer hypotensive events [9]. Protocol-based sedation outperformed daily interruption; nurse-implemented algorithms resulted in less agitation and shorter ventilation than interruption alone [11], and protocolized sedation did not benefit from added interruption [2]. Real-world practice showed high opioid use but inconsistent sedation assessment across centers [1].

Results on Delirium Outcomes

Non-opioid α₂-agonist sedation showed consistent advantages in delirium reduction. Dexmedetomidine significantly decreased delirium incidence in postoperative elderly patients [3] and increased delirium-free days in multiple trials [4,5,8]. In agitated delirium, adjunctive dexmedetomidine accelerated symptom resolution and increased delirium-free time [7]. Antipsychotics showed mixed effects; haloperidol had no impact on delirium duration but reduced agitation and rescue benzodiazepine use [10].

Results on Recovery and Clinical Outcomes

Dexmedetomidine was associated with shorter time to extubation compared with midazolam and propofol in several studies [4,5,7]. However, mortality benefits were not demonstrated; in septic patients, dexmedetomidine did not reduce 28-day mortality or ventilator-free days overall, although benefits were suggested in patients with higher illness severity [6]. Protocolized sedation and antipsychotics showed neutral effects on length of stay or mortality [2,10]. Daily sedation interruption alone resulted in longer ventilation and higher mortality in one study [11]. Remimazolam provided efficient procedural recovery comparable to propofol [9].

Results on Safety and Adverse Effects

Adverse effects varied by drug. Dexmedetomidine caused predictable bradycardia and hypotension but fewer hypertensive or tachycardic episodes than conventional sedatives [4,5]. Low-dose prophylactic dexmedetomidine reduced hemodynamic instability without excess hypotension [3], and bradycardia was typically mild [6]. Remimazolam demonstrated lower hypotension rates than propofol and no organ toxicity [9]. Haloperidol was well tolerated with few serious events [10]. Protocol-based approaches did not introduce pharmacologic risks but were associated with agitation-related complications such as device removal in some cases [1,11].

Discussion

This systematic review summarizes contemporary evidence on analgesic and sedative strategies in adult intensive care units, highlighting a clear transition from traditional opioid- and benzodiazepine-heavy regimens toward multimodal, non-opioid, and protocol-guided approaches. Across the included studies, light and cooperative sedation emerged as a consistent theme associated with improved clinical trajectories, including reduced delirium, earlier liberation from mechanical ventilation, and better patient interaction without compromising safety.

Non-GABA-based sedatives, particularly alpha-2 agonists, demonstrated favorable sedation profiles characterized by greater arousability and fewer episodes of deep sedation or coma. These properties appear to support more effective neurological assessment and may facilitate participation in care processes such as spontaneous breathing trials and early mobilization. Remimazolam also showed promise as an alternative sedative, achieving adequate sedation with fewer hemodynamic disturbances in selected ICU settings. Together, these findings suggest that sedative choice plays an important role in balancing comfort, safety, and recovery.

Beyond pharmacologic selection, this review underscores the importance of how sedation is delivered. Protocolized and nurse-driven strategies were associated with more consistent sedation targeting and improved efficiency compared with approaches relying solely on daily sedation interruption. These results indicate that structured implementation, regular reassessment, and alignment of analgesia and sedation goals may be as important as the agents themselves in optimizing outcomes.

Several limitations should be acknowledged. The included studies were heterogeneous in terms of patient populations, clinical settings, sedation targets, and outcome definitions, limiting direct comparison and precluding quantitative meta-analysis. Some trials focused on specific subgroups, such as postoperative or septic patients, which may affect generalizability. In addition, long-term outcomes, including cognitive function and quality of life after ICU discharge, were not consistently evaluated.

## Conclusions

This review highlights a clear shift in ICU practice away from deep benzodiazepine-based sedation toward multimodal, non-opioid, and protocol-guided strategies. Among available agents, dexmedetomidine offers the strongest evidence for improving sedation quality, reducing delirium, and accelerating recovery while maintaining a favorable safety profile. When combined with structured pain and sedation protocols, these approaches can meaningfully improve patient outcomes and ICU efficiency. Future research should prioritize long-term cognitive outcomes, economic impact, and integration of non-pharmacologic therapies to further optimize comfort and recovery in critically ill adults.

## Appendices

Search stragery

Pubmed: 337: (("Analgesia"[Mesh] OR analgesia[tiab] OR analgesic*[tiab] OR pain management[tiab] OR multimodal analgesia[tiab] OR "Non-opioid"[tiab] OR "Opioid"[tiab] OR sedation[tiab] OR sedative*[tiab] OR "Sedation"[Mesh])  AND ("Intensive Care Units"[Mesh] OR "Critical Care"[Mesh] OR ICU[tiab] OR "intensive care"[tiab] OR "critical care"[tiab] OR "critically ill"[tiab])  AND  (adult*[tiab] OR "Adults"[Mesh])  AND  (randomized controlled trial[pt] OR controlled clinical trial[pt] OR observational[tiab] OR cohort[tiab] OR "clinical trial"[tiab] OR prospective[tiab])  AND  (pain[tiab] OR delirium[tiab] OR "Delirium"[Mesh] OR recovery[tiab] OR "length of stay"[tiab] OR "mechanical ventilation"[tiab] OR mortality[tiab])) limited to observational studies, clinical trials and Human

Cochrane: 2040: (analgesia OR analgesic* OR "pain management" OR "non-opioid" OR opioid OR sedative* OR sedation) AND ("intensive care" OR "critical care" OR ICU OR "critically ill") AND  (adult OR adults) AND  (pain OR delirium OR recovery OR "mechanical ventilation" OR mortality OR "length of stay")

Web of science: 2,593: (analgesia OR analgesic* OR "pain management" OR "non-opioid" OR opioid OR sedative* OR sedation) AND ("intensive care" OR "critical care" OR ICU OR "critically ill") AND  (adult OR adults) AND  (pain OR delirium OR recovery OR "mechanical ventilation" OR mortality OR "length of stay")

Scopus: 7,595: ( TITLE-ABS-KEY ( analgesia OR analgesic* OR "pain management" OR "non-opioid" OR opioid OR sedative* OR sedation ) AND TITLE-ABS-KEY ( "intensive care" OR "critical care" OR ICU OR "critically ill" ) AND TITLE-ABS-KEY ( pain OR delirium OR recovery OR "mechanical ventilation" OR "length of stay" OR mortality ) AND TITLE-ABS-KEY ( adult OR adults ) ) AND ( LIMIT-TO ( DOCTYPE , "ar" ) ) AND ( LIMIT-TO ( LANGUAGE , "English" ) ) AND ( LIMIT-TO ( EXACTKEYWORD , "Human" ) ) AND ( LIMIT-TO ( SRCTYPE , "j" ) )
